# Degeneracy and Marriage

**Published:** 1904-04-09

**Authors:** 


### The Hospital.

A Journal of

The Medical Sciences and Hospital Administration.

Vol. XXXVI.—No. 915 APRIL 9, 1904.

### DEGENERACY AND MARRIAGE.

Sir James Crichton Browne has just been sounding a note of alarm, we hope somewhat in excess of the actual necessities of the case, on the subject of national physical degeneration as a consequence of undesirable marriages. His communication on the subject appeared in the columns of a daily contemporary, and can hardly fail to excite considerable attention among the very numerous readers to whom that paper appeals, more especially as the first article is to be followed by a second, in the course of a few days, on the subject of remedial measures. Even in the first, these measures are not obscurely hinted at; for Sir James tells his readers that what he rightly describes as "a stringent and somewhat startling proposal," a proposal, that is, to require the sanction of a sort of medical board as a preliminary to the solemnisation or secularisation of any marriage, is being "seriously entertained in the more go-ahead States of the American Union." In Sir James's opinion, the operatives of the East End of London afford conclusive evidence of the extent to which physical degeneration has already occurred among great masses of our people. He describes them as "squads of stunted decrepit creatures, with a look of sickliness," and declares that to compare them with the crowd in Hyde Park on a Sunday Parade is to realise that there are two races in England, nearly as distinct as Hottentots and Patagonians.

Many years have passed away since Sir James Crichton Browne first did good service to his country by calling attention to the prevalence and to the probable or inevitable consequences of what he described as overstrain in schools, especially as it was likely to affect underfed children; and we cannot but think that the aspect of certain social evils, as he then regarded them, has to a certain extent coloured his subsequent studies in the same direction, and has caused physical degeneracy to assume in his eyes something more than its real proportion to the population of the country. There are, no doubt, thousands of people in the poorer parts of our great cities who do not approach even to very moderate standards of symmetry or of athleticism. But, setting aside individuals, if we use such materials as exist for the purpose of instituting a comparison between the factory population as it is to-day, and as it was before it was protected by legislation, we think there can be no doubt that, on the whole, the verdict would be one of improvement rather than of retrogression. The diminution of adult mortality in many industrial occupations is at least unquestionable; and the alarming figures cited by Sir James to show the increase in the death-rates from cancer and alcoholism require to be considered in relation to the fact that the general death-rate of the country has diminished in much greater proportion than the increase from single causes. The immediate cause of the commencement of our factory legislation was the fearful spread throughout the factory district of Manchester of epidemic disease, which made dreadful havoc among the youthful labouring population, and which must have produced an amount of physical degeneration among the survivors, the remote fruits of which we may only now be observing. Pauper children in great numbers were sent up from the agricultural districts of the south to work as "apprentices," and were not only half starved and half naked, but were kept working until they dropped from fatigue. It was not until 1819 that factory work for children under nine was prohibited, or that children between nine and sixteen were not allowed to work more than twelve hours a day; and it was not until 1802 that the law required an apprentice to be provided with two suits of clothing, one of which was to be new each year. Notwithstanding this partial protection, when Mrs. Trollope wrote the "Factory Boy" in 1840, it was illustrated by pictures of ragged and emaciated children which would now be regarded only as gross exaggerations of the truth, but which then produced no adverse comment, and were, perhaps, as influential in reforming the abuses they displayed as was "Uncle Tom's Cabin" in calling attention to the evils of slavery. On these grounds, and on many others that could be cited, we shall look forward hopefully to the report of the Royal Commission on degeneration and shall hope to learn from it that we are slowly escaping from the errors of the past, rather than that we are falling into fresh ones peculiar to our own age and generation. Even if the latter aspect of the case should unfortunately be shown to be the correct one, we doubt whether the establishment of a medical veto upon marriages would be likely to produce any appreciable improvement. We are inclined to think that offspring are often influenced by conditions which would not be disclosed by medical examination of the parents; and moreover, as an important contribution to the inquiry, we should like to compare the percentage of manifest degenerates in the community with the percentages of illegitimate births. Of these we have at hand no recent statistics; but the return of the Registrar-General for 1888 gave them as 4·6 per cent. of the total, and this proportion was much exceeded in certain localities. The proportion in Hereford was 8·5 per cent., in Shropshire it was 8·0 per cent., and in Cumberland, Norfolk, and North Wales it ranged between 7·0 and 8·0, while in all these places it was something in excess of the proportion for the preceding decade. It would not surprise us to learn that these births supply an appreciable proportion of any degeneracy which exists.

